# Profiling of aminoxyTMT-labeled bovine milk oligosaccharides reveals substantial variation in oligosaccharide abundance between dairy cattle breeds

**DOI:** 10.1038/s41598-019-41956-x

**Published:** 2019-04-02

**Authors:** Randall C. Robinson, Nina A. Poulsen, Emeline Colet, Chloe Duchene, Lotte Bach Larsen, Daniela Barile

**Affiliations:** 1 Department of Food Science and Technology, University of California-Davis, Davis, California 95616 United States; 2 Department of Food Science, Aarhus University, Blichers Allé 20, DK-8830 Tjele, Denmark; 3 Foods for Health Institute, University of California-Davis, Davis, California 95616 United States

## Abstract

Free milk oligosaccharides are bioactive molecules that function as prebiotics and prevent infections that commonly afflict developing infants. To date, few publications have examined the factors affecting bovine milk oligosaccharide production among cattle in the dairy industry. Here we have applied a high-throughput isobaric labeling technique to measure oligosaccharide abundances in milk collected from Danish Holstein-Friesian and Jersey dairy cattle by liquid chromatography-mass spectrometry. With a total of 634 milk samples, this collection represents the largest sample set used for milk oligosaccharide profiling in the current literature. This study is also the first to use isobaric labeling for the purpose of measuring free oligosaccharides in a real sample set. We have identified 13 oligosaccharides that vary significantly by breed, with most structures being more abundant in the milk of Jersey cattle. The abundances of several oligosaccharides were increased in second-parity cows, and correlations between the abundances of oligosaccharide pairs were identified, potentially indicating similarities in their synthetic pathways. Fucosylated oligosaccharide structures were widely identified among both breeds. Improving our understanding of oligosaccharide production will aid in developing strategies to recover these compounds from processing streams and may enable their use as a functional ingredient in foods for infants and adults.

## Introduction

Milk oligosaccharides (OS) are gaining recognition as impactful bioactive compounds that can promote human health through a multitude of biological functionalities. Research over the past two decades has profiled the diverse collection of OS structures present in human milk^1,2^ and has documented their abilities to act as selective prebiotics^3–5^, modulate the immune system^6–8^, and prevent diseases common among infants, including diarrhea^9,10^ and necrotizing enterocolitis^11,12^. Bacteria found in the intestines of breast-fed infants possess the unique ensembles of genes needed to metabolize these compounds^13,14^, and human milk OS are thought to play a major role in the establishment of the infant immune system^15,16^ and gut microbiota^16–18^.

A more recent wave of studies is uncovering the potential of readily-available bovine milk OS to improve child and adult health by alleviating a variety of adverse inflammatory, metabolic and digestive conditions. Impaired gut barrier function has been linked with prevalent disorders such as diet-induced obesity^19,20^ and inflammatory bowel diseases^21,22^, and there is a need to develop therapeutic strategies to alleviate these widespread issues. Dietary supplementation with bovine milk OS has led to reduced weight gain in mouse models of diet-induced obesity^23^ and can prevent obesity-associated increases in intestinal permeability and microbial dysbiosis^23,24^. Furthermore, their effectiveness as anti-inflammatory therapeutics has been demonstrated in studies showing reduced expression of inflammatory genes and prevention of fatty liver development when taken as a synbiotic with *Bifidobacterium infantis*^25^.

In the realm of metabolism, bovine milk OS have shown promise as alleviators of the metabolic irregularities associated with undernutrition. When consuming diets supplemented with bovine milk OS, mouse and pig models of infant undernutrition demonstrated changes in metabolite profiles and improved bone and organ growth, as well as a microbially-mediated increase in lean body mass^26^. These results provide strong evidence that dietary supplementation with OS can alleviate the undernourished phenotype. Furthermore, the bovine milk OS pool has many OS structures in common with human milk^27,28^ and possesses similar prebiotic properties *in vivo*^25^. Given these diverse and valuable bioactivities, there is substantial interest in making milk OS more readily available in both infant formula and therapeutic foods for adults.

OS from human and bovine milk typically consist of a lactose core at the reducing end, which is elongated by the monosaccharides galactose (Gal), N-acetylglucosamine (GlcNAc), N-acetylgalactosamine (GalNAc, bovine only), fucose (Fuc), N-acetylneuraminic acid (NeuAc), and N-glycolylneuraminic acid (NeuGc, bovine only, found in trace abundance)^16,27,29^. The collection of bovine milk OS has been profiled by liquid chromatography–mass spectrometry (LC-MS) in several studies^27,30,31^. A recent study examined the milk OS content of 20 samples from Holstein-Friesian and Jersey cattle and found that the Jersey milk contained greater amounts of sialylated and fucosylated OS^32^. Given that Holstein-Friesian and Jersey are two of the most commonly used breeds for milk production, we have designed a comprehensive study to further investigate the milk OS content of these cattle breeds to better understand how factors such as breed and milk yield may influence variation in the milk OS profile. We have measured relative quantities of OS in a large collection of milk samples using LC-MS and isobaric labeling, an analytical technique that improves instrumental throughput for large sample sets by allowing samples to be multiplexed prior to analysis by mass spectrometry^33^. Building our understanding of variables that affect OS abundances in milk and its processing side-products will enable OS to be recovered more efficiently from industrial processing streams for use in infant formulas and therapeutic foods for children and adults.

## Methods

### Sample Collection

Milk samples were collected from 334 Danish Holstein-Friesian and 300 Danish Jersey cows under the Danish-Swedish Milk Genomics Initiative. The samples came from 40 total herds and were collected from cows consuming a total mixed ration during the indoor housing period. All samples were from the morning milking and were transported to the lab on ice, where they were frozen at −40 °C. Sample collection was designed to maximize genetic variability among the cattle, while ensuring that all cows were within parity 1–3 and were in mid-lactation. The complete sample collection process has been described previously in greater detail^34^.

### Oligosaccharide extraction and isobaric labeling

Oligosaccharides were extracted and labeled with isobaric reagents as described previously^33^. Briefly, milk aliquots of 400 µL were combined with an equal volume of Milli-Q water and skimmed at 4,000 × g and 4 °C for 30 min. The skim milk was combined with two volumes of cold ethanol and placed in a −30 °C freezer for 1 hour to precipitate proteins. Samples were then centrifuged at 4,000 × g and 4 °C for 30 min, and the supernatants were transferred to new tubes and dried by centrifugal evaporation.

The samples were re-dissolved in 300 µL Milli-Q water and purified by microplate C18 solid phase extraction (SPE) to remove residual lipids, peptides, and proteins. The C18 microplates were conditioned with acetonitrile (ACN) and equilibrated with water. Samples were loaded, and the plate was washed with three column volumes of water (600 µL total) to ensure complete elution of the OS. All liquid that eluted from the plate during and after sample loading was collected. Acetonitrile and trifluoroacetic acid (TFA) were added to these solutions to adjust the solvent composition to 2% ACN/0.1% TFA, and the samples were then purified by porous graphitized carbon (PGC) SPE to eliminate salts and reduce the lactose content. The PGC SPE microplates were conditioned with 80% ACN/0.1% TFA and equilibrated with 2% ACN/0.1% TFA. Samples were loaded, and the microplate wells were each washed with 1.2 mL 2% ACN/0.1% TFA. The OS were then eluted with three column volumes (600 µL total) 40% ACN/0.1% TFA and dried by centrifugal evaporation.

Aliquots of 7.5% of each sample extract were labeled with aminoxy tandem mass tag (TMT) reagents using the manufacturer’s protocol (ThermoFisher Scientific, Waltham, MA). Since each multiplexed set has a maximum of six available reporter ion channels and the study included over 600 total samples, the samples were multiplexed using a recently-published strategy^33^ in which each multiplexed vial contains five TMT-labeled samples and one TMT-labeled OS standard mixture that is used to normalize the TMT reporter ion intensities. The bovine milk OS powder used as the standard mixture was produced according to processing techniques described previously^35^ and was extracted using the C18 and PGC SPE procedures described above. The multiplexed sample sets were dried and re-dissolved in 3% ACN for LC-tandem-MS (LC-MS/MS) analysis.

### Relative quantification by LC-MS/MS

Samples were analyzed on an Agilent 6520 Accurate-Mass Q-TOF LC/MS system, equipped with a Chip Cube interface. Chromatographic separation was performed on a PGC high-performance LC chip, which contained a 40 nL loading column and a 75 µm × 43 mm analytical column, both packed with 5 µm particles. Instrumental parameters for chromatographic separation and OS analysis by MS have been described previously^33^. Ionization source voltage was varied from 1825–1890 V to maintain a stable solvent spray.

### OS identification and relative quantification

OS identities were confirmed by examination of the MS/MS spectra using Agilent MassHunter B.06.00. A list of identified OS and their respective m/z values and retention times were entered into SimGlycan 5.42 Enterprise Edition^36^ as a custom library. The program’s “High Throughput Search and Score” feature was used to identify OS based on their precursor mass and retention time. Reporter ion abundances were summed for each OS and then normalized to the corresponding internal standard channel. Error tolerances used for identification of labeled OS and their fragments in the mass spectral data were 10 ppm and 0.025 Da for precursor and reporter ions, respectively.

### Statistical analysis

Wilcoxon-Mann-Whitney tests were used in R (version 3.5.0) to compare the abundance of each OS between breeds due to non-normal distributions of OS abundances. Within each breed, differences in the abundance of each OS by parity were identified with a Kruskal-Wallis test, followed by pairwise Kruskal-Wallis comparisons. For each set of pairwise comparisons, the overall α-value was adjusted to 0.05 using the “pairw.kw” function in R. Principal component analysis and calculation of Pearson’s correlation values were conducted in R. Pearson correlation figures and their significances (α = 0.01) were generated using the R-package corrplot^37^. When generating Pearson correlation figures and the principal component analysis plot, zeroes were entered for any OS which were below the level of quantification.

### Use of experimental animals

Experimental protocols for sample collection were approved by the National Guidelines for Animal Experimentation and the Danish Animal Experimental Ethics Committee. All experiments were performed in accordance with relevant guidelines and regulations.

## Results

### OS abundances vary by breed

**Figure 1:**
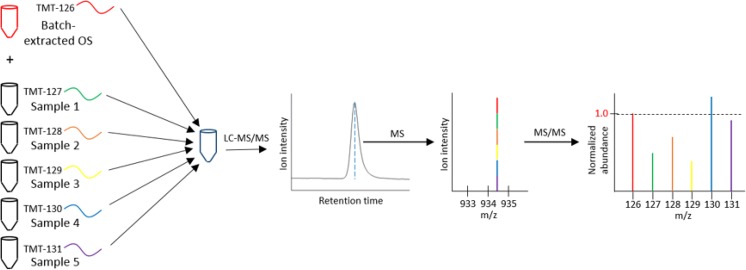
Multiplexing and LC-MS/MS analysis of milk OS. Each multiplexed vial is spiked with a collection of batch-extracted milk OS, labeled with the TMT^6^-126 reagent. Groups of five samples are labeled with the remaining isobaric reagents and analyzed by LC-MS/MS. Reporter ion signals in the tandem spectra are normalized to the signal originating from the corresponding OS in the batch-extracted mixture.

Milk from Holstein-Friesian and Jersey cattle showed a high degree of similarity in the OS compositions and structures present in the sample sets. Abundances for 15 OS were measured in both breeds. In order to feasibly analyze this large sample set by MS, we have implemented a recently-developed isobaric labeling technique for milk OS^33^ that allows samples to be multiplexed prior to MS analysis. Following data acquisition, the abundance of each OS in each sample is normalized to a reference signal originating from a TMT-labeled OS standard mixture that is spiked into each multiplexed vial in identical quantities. Analyte abundances measured across the numerous multiplexed sets are therefore comparable. Figure 1 provides a graphical summary of the analytical method. The complete dataset is available as Supplementary Tables S1, S2.

**Figure 2:**
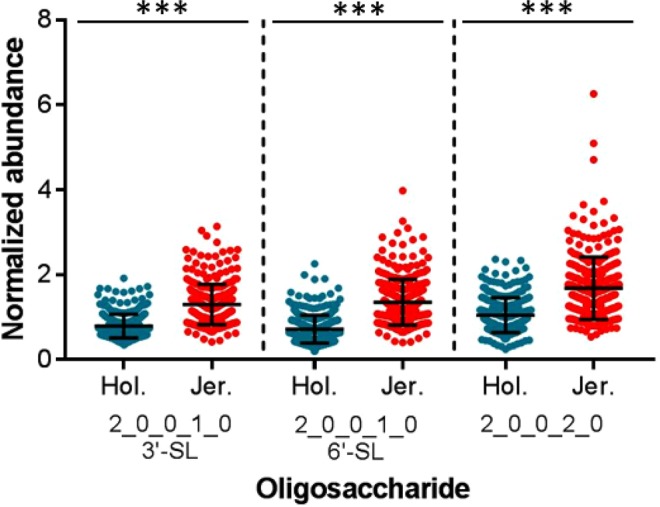
Abundances of the acidic (sialic-acid containing) milk oligosaccharides. The oligosaccharides are represented by their monosaccharide compositions, denoted as the number of each monosaccharide type present in the compound (Hex_HexNAc_Fuc_NeuAc_NeuGc). Error bars show mean ± standard deviation. Statistically significant differences in oligosaccharide abundances were determined with Wilcoxon-Mann-Whitney tests. 3′-SL, 3′-sialyllactose; 6′-SL, 6′-sialyllactose; ***p < 2.2 × 10^−16^.

Acidic (sialic acid-containing) OS are one of the most abundant categories of bovine milk OS, with a total abundance similar to or greater than that of the neutral compounds^31,38^. We identified the OS isomer pair 3′-sialyllactose (3′-SL) and 6′-sialyllactose (6′-SL) normally present in bovine milk, as well as the OS with composition 2 Hex 2 NeuAc. Each of these compounds was significantly more abundant on average (p < 2.2 × 10^−16^ for each OS) in the milk of the Jersey cattle (Fig. 2). The breeds showed similar degrees of cow-to-cow variation in acidic OS abundances, with coefficients of variation (CVs) ranging from 35–46% for Holstein-Friesian cattle and 37–44% for Jersey cattle. OS containing the NeuGc monosaccharide were not identified in the sample set.

**Figure 3:**
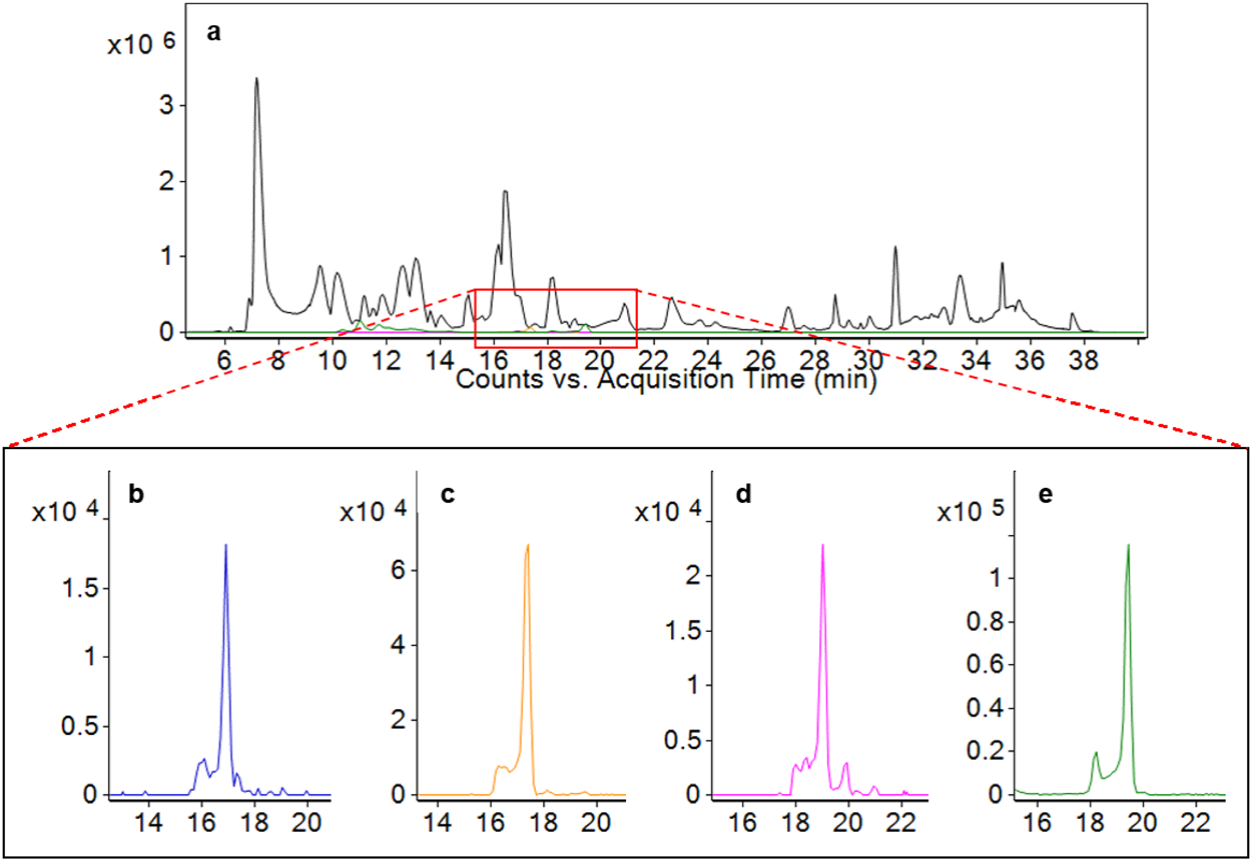
Base peak chromatogram of a typical multiplexed set of Jersey milk samples (**a**, black trace). Extracted ion chromatograms are shown below for the fucosylated oligosaccharides 4 Hex 4 HexNAc 1 Fuc (**b**), 5 Hex 4 HexNAc 1 Fuc (**c**), 4 Hex 5 HexNAc 1 Fuc (**d**), and 3 Hex 6 HexNAc 1 Fuc (**e**).

**Figure 4:**
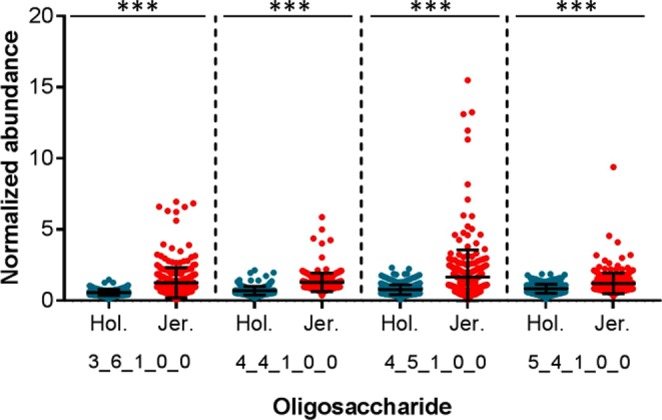
Abundances of the fucosylated milk oligosaccharides. The oligosaccharides are represented by their monosaccharide compositions, denoted as the number of each monosaccharide type present in the compound (Hex_HexNAc_Fuc_NeuAc_NeuGc). Error bars show mean ± standard deviation. Statistically significant differences in oligosaccharide abundances were determined with Wilcoxon-Mann-Whitney tests. ***p < 2.2 × 10^−16^.

Four fucosylated OS were identified with a high degree of consistency in milk from both breeds. These OS provided relatively strong signals in the MS data (Fig. 3), likely due to the improvement in ionization efficiency provided by the TMT reagents^33,39^. The exact structures/linkages of these OS are currently unknown, but we have deduced their monosaccharide compositions to be 3 Hex 6 HexNAc 1 Fuc, 4 Hex 4 HexNAc 1 Fuc, 4 Hex 5 HexNAc 1 Fuc, and 5 Hex 4 HexNAc 1 Fuc. As with the acidic OS, the mean abundance of each fucosylated OS was significantly higher (p < 2.2 × 10^−16^ in every case) in the Jersey breed (Fig. 4). The Jersey milk also demonstrated much greater cow-to-cow variability in fucosylated OS abundance. Coefficients of variation for these OS in the Jersey breed ranged from 50–115%, compared with 36–46% among Holstein-Friesian cattle. As Fig. 4 illustrates, for each OS a small subset of Jersey cows produced much greater quantities of these compounds than their within-breed counterparts. Cows that produced the highest quantities of one of these fucosylated OS were typically also excellent producers of the other three.

**Figure 5:**
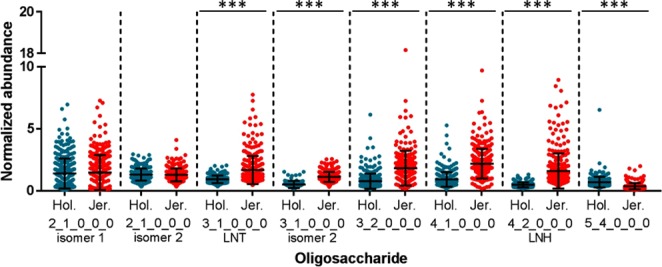
Abundances of the neutral, non-fucosylated milk oligosaccharides. The oligosaccharides are represented by their monosaccharide compositions, denoted as the number of each monosaccharide type present in the compound (Hex_HexNAc_Fuc_NeuAc_NeuGc). Error bars show mean ± standard deviation. Statistically significant differences in oligosaccharide abundances were determined with Wilcoxon-Mann-Whitney tests. LNT, lacto-N-tetraose; LNH, lacto-N-hexaose; ***p < 2.2 × 10^−16^.

Eight other neutral, non-fucosylated OS were identified with consistency in the samples, ranging from trisaccharides to larger OS, reaching a degree of polymerization of nine monosaccharide units. The majority of these neutral compounds had significantly greater average abundances in the milk of Jersey cattle, with p < 2.2 × 10^−16^ for all OS with significant differences between breeds. The OS with composition 5 Hex 4 HexNAc was unique in its significantly lower abundance within the Jersey breed (p < 2.2 × 10^−16^). The two OS isomers with compositions of 2 Hex 1 HexNAc were the only compounds without significant differences in abundance between breeds, with p = 0.263 (isomer 1) and p = 0.531 (isomer 2). The Jersey breed again showed greater animal-to-animal variation in the abundances of these OS (Fig. 5), with coefficients of variation ranging from 32–86% (Holstein) and 36–95% (Jersey). Supplementary Table S3 provides a complete list of p- and N-values for each comparison of OS abundance by breed.

To aid in summarizing the breed-level comparisons, we performed a three-dimensional principal component analysis (Supplementary Fig. S1), which represents 60.9% of the variation among the data collected. The fucosylated OS, which showed especially high variation in the Jersey breed, contributed heavily to the second principal component. In general, greater dispersion of the Jersey samples is evident, which agrees well with the individual OS measurements.

### Oligosaccharide abundance increases at second parity

The animals used in this study were all within first to third parity (i.e., milk was collected following the first, second, or third pregnancy). Cows at second parity possessed significantly greater quantities of several OS in comparison to first parity cows. In the Holstein-Friesian breed, the acidic OS 3′-SL and 6′-SL were significantly more abundant in the second parity milk compared to first parity milk (p = 0.00305 and p = 0.00014, respectively), as well as the neutral OS lacto-N-tetraose (p = 0.00139) and the OS with compositions 5 Hex 4 HexNAc (p = 0.000236) and 2 Hex 1 HexNAc (isomer 2, p = 0.007038). By comparison there were fewer differences between second and third parity milk (Table 1). In the Jersey samples, the OS 3′-SL (p = 0.002896), 6′-SL (p = 0.003073), lacto-N-tetraose (p = 0.003487), 2 Hex 1 HexNAc (isomer 2, p = 0.008072), 3 Hex 2 HexNAc (p = 0.008192), and 4 Hex 1 HexNAc (p = 0.027937) were all significantly more abundant in second parity samples compared to those of first parity (Table 2). Again, we identified fewer significant differences when comparing first and third parity samples. Supplemental Tables S4, S5 provides the p-values for each Kruskal-Wallis and pairwise comparison test.

**Table 1:** Mean relative abundances (±standard deviation) of OS that vary significantly by parity in the Holstein-Fresian breed.

Oligosaccharide	Mean parity 1 abundance	Mean parity 2 abundance	Mean parity 3 abundance
3′-sialyllactose	0.741 (±0.270)^a^	0.840 (±0.271)^b^	0.819 (±0.302)^ab^
6′-sialyllactose	0.626 (±0.257)^a^	0.797 (±0.353)^b^	0.800 (±0.375)^b^
Lacto-N-tetraose	0.882 (±0.284)^a^	1.024 (±0.322)^b^	0.932 (±0.283)^ab^
2_1_0_0_0 (isomer 2)	1.208 (±0.430)^a^	1.438 (±0.497)^b^	1.390 (±0.564)^ab^
5_4_0_0_0	0.652 (±0.521)^a^	0.745 (±0.283)^b^	0.768 (±0.348)^b^

OS without common names are represented by their monosaccharide compositions, denoted as the number of each monosaccharide type present in the compound (Hex_HexNAc_Fuc_NeuAc_NeuGc). Across each row, abundance values that share the same superscript letter are not significantly different at α = 0.05 using pairwise Kruskal-Wallis comparisons. Exact p-values for each pairwise comparison are provided as Supplementary Table 3.

**Table 2:** Mean relative abundances (±standard deviation) of OS that vary significantly by parity in the Jersey breed.

Oligosaccharide	Mean parity 1 abundance	Mean parity 2 abundance	Mean parity 3 abundance
3′-sialyllactose	1.184 (±0.362)^a^	1.445 (±0.566)^b^	1.351 (±0.508)^ab^
6′-sialyllactose	1.222 (±0.441)^a^	1.454 (±0.580)^b^	1.504 (±0.0619)^b^
Lacto-N-tetraose	1.560 (±1.167)^a^	1.808 (±1.152)^b^	1.801 (±0.990)^b^
2_1_0_0_0 (isomer 2)	1.205 (±0.419)^a^	1.451 (±0.622)^b^	1.281 (±0.538)^ab^
3_2_0_0_0	1.608 (±0.867)^a^	2.162 (±1.981)^b^	1.866 (±1.269)^ab^
4_1_0_0_0	1.995 (±0.964)^a^	2.482 (±1.476)^b^	2.242 (±1.105)^ab^
Lacto-N-hexaose	1.469 (±1.478)^a^	1.752 (±1.282)^b^	1.727 (±1.413)^ab^

OS without common names are represented by their monosaccharide compositions, denoted as the number of each monosaccharide type present in the compound (Hex_HexNAc_Fuc_NeuAc_NeuGc). Across each row, abundance values that share the same superscript letter are not significantly different at α = 0.05 using pairwise Kruskal-Wallis comparisons. Exact p-values for each pairwise comparison are provided as Supplementary Table 4.

### Correlations among OS abundances may provide insights into synthetic pathways

**Figure 6:**
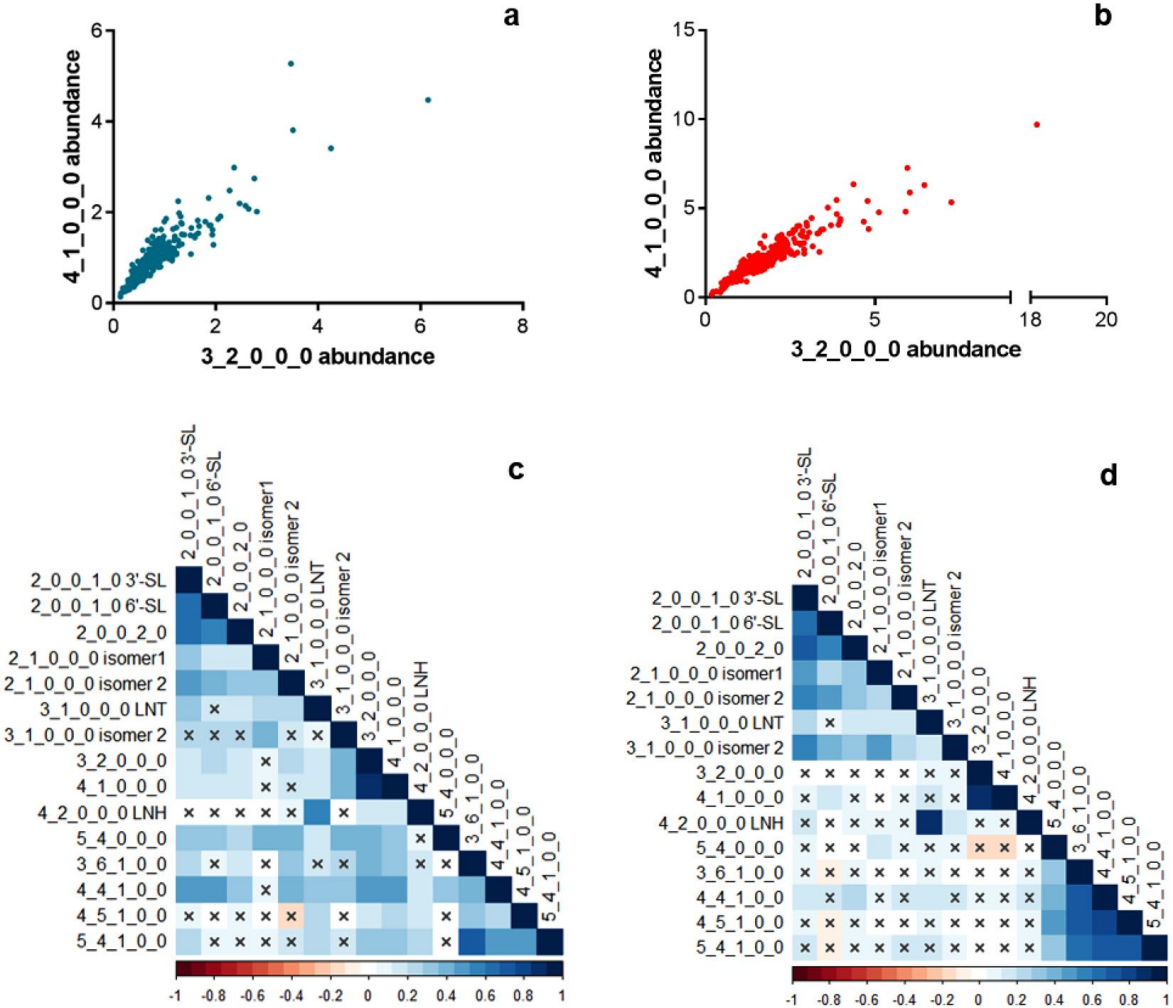
Correlations among oligosaccharide pairs. The oligosaccharide compositions 3 Hex 2 HexNAc and 4 Hex 1 HexNAc showed strong correlations in both Holstein-Fresian (**a**, r = 0.936) and Jersey (**b**, r = 0.937) samples. Correlations for all oligosaccharide pairs are shown in parts **c** (Holstein-Fresian) and **d** (Jersey) as heat maps. Correlations marked with a cross were not significant based on the F-test (P > 0.01). The oligosaccharides are represented by their monosaccharide compositions, denoted as the number of each monosaccharide type present in the compound (Hex_HexNAc_Fuc_NeuAc_NeuGc).

We identified several correlations among the OS abundances of both breeds, the strongest of which exists between the OS with compositions 4 Hex 1 HexNAc and 3 Hex 2 HexNAc (Pearson’s r of 0.936 and 0.937 for Holstein-Friesian and Jersey, respectively). Figure 6a,b shows these correlations graphically for each breed. Another noteworthy correlation exists between the OS pair lacto-N-tetraose and lacto-N-hexaose in Jersey milk (r = 0.910). Moderate correlations (0.6 ≤ r ≤ 0.8) were seen among several fucosylated OS pairs from both breeds. Correlations for all OS pairs are shown pictorially in heat map format in Fig. 6c,d and are provided numerically as Supplementary Tables S6 (Holstein-Friesian) and S7 (Jersey). Correlations marked with a cross were not significant based on the F-test (P > 0.01).

Since milk yield data was collected for the cattle used in the study (recorded as liters collected during the morning milking), we have also explored correlations between milk yield and the abundance of each OS within each breed. However, substantial correlations did not exist in either breed (Supplementary Table S8).

## Discussion

The ability to conduct broad genetic and phenotypic profiling of dairy cattle has substantially impacted the dairy industry over the past decade. Knowledge of genetic and phenotypic variations that are relevant for milk production in combination with low-cost genotyping products allows dairies to select for desirable traits and improve the genetic quality of their cattle more rapidly than was previously possible^40^. Although other milk traits have been thoroughly investigated in dairy cattle, milk oligosaccharide profiling has previously been conducted with relatively small sample sets. In order to better characterize the diversity in milk OS content within the dairy industry, we have profiled a collection of milk samples from a total of 634 mid-lactation Holstein-Friesian and Jersey dairy cattle. The Holstein-Friesian breed is widely used among dairies and is known for its high milk yield^41,42^. Jersey cows produce comparatively less milk volume, but their milk has attracted attention due to its higher concentration of total protein and fat^42,43^. In light of the known bioactivities of bovine milk OS and the recently-developed processing strategies for OS isolation from dairy side streams^44,45^, it is likely that the ability to select for desirable milk OS compositions among these dairy breeds will be of future value to the dairy industry.

With a total of 634 milk samples, this collection represents the largest sample set used for milk OS profiling in the current literature. This study is also the first to use isobaric labeling for the purpose of measuring relative analyte abundances in a large sample set. Relative quantification by isobaric labeling has previously been implemented only with smaller sample sets due to the fact that the reagent kits typically contain 10 or fewer label variants. We have developed an instrumental technique to analyze larger collections of TMT-labeled milk OS and have characterized the accuracy and reproducibility to be on par with other LC-MS/MS-based relative quantification strategies^33^. This ability to analyze samples as mixtures substantially increased instrumental throughput and improved the feasibility of measuring individual OS abundances in such a large sample set.

In comparing the Danish Holstein-Friesian and Jersey breeds, we have found that Jersey milk contains a greater mean abundance of 12 of the 15 OS identified in the sample set. Among bovine milk OS, 3′-SL is typically the most abundant compound^46,47^, and we have measured significantly more of this OS in the Jersey breed (mean normalized abundance of 0.79 ± 0.28 Holstein-Friesian vs. 1.30 ± 0.48 Jersey, p < 2.2 × 10^−16^). 3′-Sialyllactose, in addition to the identified 6′-SL and lacto-N-tetraose, has been characterized as having prebiotic properties^3,48,49^. This result in conjunction with the data for the remaining OS strongly suggest that milk from the Jersey breed contains a greater total OS concentration. Only one OS, with composition 5 Hex 4 HexNAc, was significantly more abundant in the Holstein-Friesian samples (p < 2.2 × 10^−16^). In light of the much higher abundance of fucosylated OS in the Jersey milk, it is intriguing to consider the possibility that the OS 5 Hex 4 HexNAc may serve as a precursor to the OS 5 Hex 4 HexNAc 1 Fuc. Increased fucosylation of existing OS structures in Jersey cows would explain their anomalously lower content of the OS 5 Hex 4 HexNAc, but substantial further work would be needed to validate this hypothesis. The Jersey breed was also notable for its much greater variation in OS abundances between animals. Though the reasons for this variability are unclear, we explored the possibility that a greater total milk yield was correlated with a more dilute milk OS ensemble. All correlations coefficients were rather weak (−0.3 < r < 0.3), indicating that OS abundance appears to vary independently of milk output.

A study by Sundekilde *et al*. examined a subset of samples from the same cattle breeds and produced similar findings, with the acidic and fucosylated OS being more abundant in the Jersey breed. One notable exception is that some smaller, neutral OS, including the composition 2 Hex 1 HexNAc, were more abundant in the Holstein-Friesian milk of the past study^32^. We have identified two isomers of composition 2 Hex 1 HexNAc, and interestingly, found that they were the only two OS which did not differ in abundance between breeds. However, the isomer with composition GalNAc(α1–3)lactose (identity confirmed by comparison with an analytical standard) did show substantial variation within each breed, with some samples containing ~4x greater abundance than the breed mean (Fig. 5).

One of the major differences between human and bovine milk OS profiles has been in the content of fucosylated OS. Quantitative studies of human milk have measured fucosylated OS concentrations of 1.8–15.8 g/L^49–53^, while fucosylated structures have been absent from all but a few bovine milk OS profiling studies^27,32,35,54^. Four studies in the past decade have identified high-molecular weight fucosylated bovine OS^27,32,33,35^, and we have identified four of these structures with consistency. These are among the largest OS known to exist in bovine milk, both in terms of molecular weight and number of monosaccharide residues, and they closely resemble the compositions of multi-functional OS in human milk. Numerous studies have associated fucosylated human milk OS with reduced incidence of bacterial infections^10,55,56^ which makes these bovine structures relevant in the context of tailoring dairy ingredients for infant formula production. Analytical techniques that can reliably identify OS structures are typically a prerequisite to further structural analysis, linkage determination, absolute quantification, and functional testing. Therefore, TMT labeling and similar OS derivatization techniques that improve ionization efficiency may be of value in further characterizing the structures and biological relevance of these fucosylated compounds.

Among the noteworthy trends identified in the study include the finding that OS abundances generally increase from first to second parity. Similar findings for sow milk OS were recently published by Wei *et al*., who have shown that numerous structures were significantly more abundant in the colostrum and transitional milk of pigs who had given birth multiple times in comparison to those giving birth for the first time^57^. The OS 6′-SL, which varied with parity in sow milk, also varied with parity in Holstein-Friesian and Jersey milk. Parity-associated variations in OS abundance have also been observed in human milk^58^. The reasons for these parity-related variations are difficult to pinpoint at this time, since little is known about the synthetic processes of free milk OS. However, it is known that numerous other factors, including bovine milk yield^59^ and protein isoform abundance^60^, as well as goat milk lactose content^61^, vary with parity. Therefore, we suspect that the increased abundance of certain OS in second parity milk is the result of underlying metabolic changes in the mammary gland.

Additionally, we have identified numerous correlations between the abundances of different OS structures in the cattle breeds. The correlation between the OS pair lacto-N-tetraose and lacto-N-hexaose in the Jersey samples is noteworthy because these OS share the type-1 core structure (Gal(β1–3)GlcNAc(β1–3)lactose)^62^ and are likely to follow similar synthetic pathways. The strongest correlation, which existed between the OS compositions 3 Hex 2 HexNAc and 4 Hex 1 HexNAc, was recently reported by Liu *et al*.^63^. Although structures for the 4 Hex 1 HexNAc composition have been determined^27,64^, the structure of the 3 Hex 2 HexNAc OS has not been completely characterized, making the reasons for this correlation difficult to elucidate. However, we hypothesize that the strong correlation between these OS indicates similarities in their synthetic pathways and linkages. Similarly, the moderate correlations identified among some fucosylated OS pairs may result from a shared collection of fucosyltransferase enzymes participating in OS synthesis. In humans, it is well-characterized that the FUT2 gene, encoding an α1–2 fucosyltransferase, influences the synthesis of OS with α1–2-linked fucose residues^16^. Based on the correlations among the fucosylated OS, we hypothesize that fucosylation of bovine milk OS may also be similarly influenced by a small number of fucosyltransferases. Although the structures of the majority of bovine milk OS and the specific enzymes which assemble these structures are not entirely elucidated, a past study by Wickramasinghe *et al*. has identified a multitude of candidate synthetic enzymes through bovine milk transcriptomes^65^. Further structural characterization of these OS will aid in identifying their respective synthetic enzymes and pathways, which may enable a greater ability to harness genetic and environmental variations to improve milk OS content for novel therapeutic applications.

## Conclusion

Emerging evidence that bovine milk OS possess unique bioactivities and are readily available in dairy processing streams may lead to the commoditization of these compounds in the near future. In combination with suitable large-scale isolation techniques, the ability to phenotypically profile large quantities of milk samples improves the practicality of pursuing industrial OS production. In this study, we have used high-throughput methodology to profile a large collection of dairy milk samples and have identified numerous factors associated with variations in bovine milk OS abundance. On average, most OS were substantially more abundant in the milk of Jersey cattle relative to Holstein-Friesian. However, the Jersey breed demonstrated much greater cow-to-cow variability. In each breed, numerous OS structures had increased abundances in second parity milk relative to that of the first parity. Furthermore, we have confirmed that fucosylated OS structures are prevalent among the studied cattle populations and have identified a technique by which these compounds can be more reliably detected by MS analysis for further characterization and functional testing.

## Supplementary information

Supplementary Figure S1

Supplementary Tables S1-S8

## Data Availability

All data generated or analysed during this study are included in this published article (and its Supplementary Information Files).
