# Supplementary material for: Profiling of aminoxyTMT-labeled bovine milk oligosaccharides reveals substantial variation in oligosaccharide abundance between dairy cattle breeds
Source: Sci Rep. 2019 Apr 2;9:5465. doi: 10.1038/s41598-019-41956-x (PMC6445284; doi:10.1038/s41598-019-41956-x)
Supplement: Supplementary file 1 — Supplementary Figure S1 [file 41598_2019_41956_MOESM1_ESM.docx]

Profiling of aminoxyTMT-labeled bovine milk oligosaccharides reveals substantial variation in oligosaccharide abundance between dairy cattle breeds

Randall C. Robinson,^1^ Nina A. Poulsen,^2^ Emeline Colet,^1^ Chloe Duchene,^1^ Lotte Bach Larsen,^2^ and Daniela Barile^1,3,*^

^1^Department of Food Science and Technology, University of California-Davis, Davis, California 95616, United States

^2^Department of Food Science, Aarhus University, Blichers Allé 20, DK-8830 Tjele, Denmark

^3^Foods for Health Institute, University of California-Davis, Davis, California, 95616, United States

*dbarile@ucdavis.edu


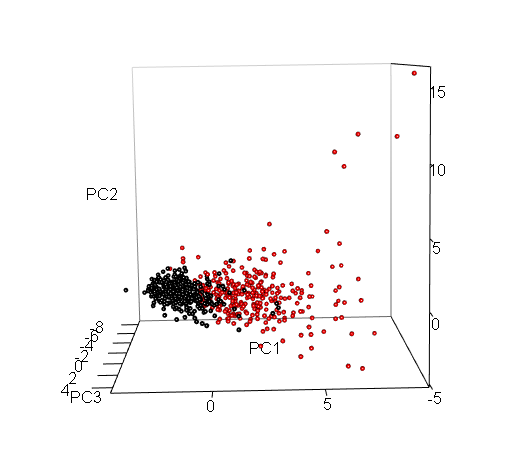


**Supplementary Figure S1.** Principal component analysis of relative oligosaccharide abundances, representing 60.9% of the variation in the dataset. The Jersey breed (red spheres) shows a greater dispersion among samples than the Holstein breed (black spheres), which is consistent with the individual oligosaccharide measurements.
